# CIGAF—a database and interactive platform for insect-associated trichomycete fungi

**DOI:** 10.1093/database/baad038

**Published:** 2023-05-23

**Authors:** Shalini Chaudhary, Yibing Wu, Doug Strongman, Yan Wang

**Affiliations:** Department of Ecology and Evolutionary Biology, University of Toronto, 25 Willcocks St, Toronto, Ontario M5S 3B2, Canada; Department of Biological Sciences, University of Toronto Scarborough, 1265 Military Trail, Toronto, Ontario M1C 1A4, Canada; Halifax, Nova Scotia B3L 1S2, Canada; Department of Ecology and Evolutionary Biology, University of Toronto, 25 Willcocks St, Toronto, Ontario M5S 3B2, Canada; Department of Biological Sciences, University of Toronto Scarborough, 1265 Military Trail, Toronto, Ontario M1C 1A4, Canada

## Abstract

Trichomycete fungi are gut symbionts of arthropods living in aquatic habitats. The lack of a central platform with accessible collection records and associated ecological metadata has limited ecological investigations of trichomycetes. We present CIGAF (short for Collections of Insect Gut–Associated Fungi), a trichomycetes-focused digital database with interactive visualization functions enabled by the R Shiny web application. CIGAF curated 3120 collection records of trichomycetes across the globe, spanning from 1929 to 2022. CIGAF allows the exploration of nearly 100 years of field collection data through the web interface, including primary published data such as insect host information, collection site coordinates, descriptions and date of collection. When possible, specimen records are supplemented with climatic measures at collection sites. As a central platform of field collection records, multiple interactive tools allow users to analyze and plot data at various levels. CIGAF provides a comprehensive resource hub to the research community for further studies in mycology, entomology, symbiosis and biogeography.

## Introduction

Freshwater ecosystems around the world contain assemblages of insects, including mosquitoes, midges, blackflies, mayflies, stoneflies and others, which spend their early life stages growing and feeding in aquatic environments. Within the gut of these larval insects lives a group of microbial fungal symbionts, historically known as the trichomycetes, which are obligately associated with their hosts (1, 2).

The trichomycete fungi are globally distributed, holding an early-diverging placement on the fungal tree of life (3–5). The symbiotic relationships between trichomycetes and aquatic insects are often regarded as commensals and presumably initiated over 200 million years ago (6). Trichomycete fungi currently consist of three orders: Asellariales, Harpellales and Orphellales, all within the subphylum Kickxellomycotina (Zoopagomycota) (7, 8). The identification and collections of trichomycete fungi require the sampling of colonized larval insect hosts from aquatic ecosystems. Typically, this works by disturbing rocks, sediment or vegetation within the target body of water and rapidly collecting dislodged insects with a mesh net held immediately downstream (kick sampling) (9). The collected insects are dissected with fine forceps and needles such that the insect’s gut lining is flattened or cut open, allowing the fungi within them to be visualized and identified with taxonomic keys (10). The efforts to study trichomycetes started nearly a century ago, with geographic sampling range, intensity of collection and documentation increasing over time, generating a large number of collection records across the globe (7, 11–14).

Despite the wealth of trichomycete collection literature, there is no unified resource to facilitate the exploration of their distribution and associated collection data. Presently, trichomycete-specific databases are limited to taxonomic descriptions and interactive keys used for specimen identification (10). Existing collection-focused databases, such as the Global Biodiversity Information Facility (GBIF), contain collection site maps but lack information about host association, collection site climate and other informative ecological parameters (15). Furthermore, these resources provide limited data visualization functionalities, forcing researchers to independently compile collection metadata using code-based tools for data analysis and visualization. This has limited the array of questions that can be accessibly explored by the research community.

Here, we present the CIGAF, a database for the Collections of Insect Gut–Associated Fungi, including 3120 collection records worldwide for trichomycetes since the first public record in 1929 (12). The CIGAF is designed to address the need for an accessible trichomycetes collection resource. It has an easy-to-use interface with data visualization tools that enable the exploration of published records. CIGAF offers several benefits for ecological research on insect gut–associated fungi and promotes related hypothesis development that initiates global-scale questions in terms of insect–fungus symbiosis, biogeography and ecological niche preference. In addition, CIGAF is also designed for educational purposes to ease the data access difficulties that may impede emerging researchers in the field who are interested in aquatic insects, trichomycetes or using broader ecological data to analyze their influences on global biota. The CIGAF database can be accessed via http://cigaf.eeb.utoronto.ca. It is free to use and open to all researchers, students and members of the public who are interested in exploring trichomycete fungi and related aquatic insect hosts around the world.

## Methods

### Literature selection

A total of 214 articles were identified from Web of Science using the search phrase (Harpellales OR Harpellomycetes) OR (Asellariales OR Asellariomycetes) OR (Orphellales OR Orphellaceae) OR (Kickxellomycotina) OR (Trichomycete OR Trichomycetes) AND (record OR found OR identified OR collection OR collected OR collect OR new OR first) (16). Eighty-five papers were excluded as they did not contain original aquatic insect–associated trichomycetes collections (*N* = 75), were not in English (*N* = 3), or did not have high-quality (fully identified) specimens (*N* = 7). Occasionally, subsequent publications, with access to additional specimens, improved imaging technology or informative sequencing methods, retroactively identified previously published fungal specimens. In these cases, the new species level identification was applied to the original collection record and the original entry was included as the sole record within the database (*N* = 2). Nineteen papers that were not indexed on Web of Science were included from a private collection of historic hard copy peer-reviewed trichomycetes literature. In total, 148 peer-reviewed journal articles were included in the CIGAF database, available within the ‘Works Cited’ tab of the CIGAF website.

### Extracting data

From each of the 148 included publications, specimen collection records and associated metadata were manually curated and entered into the database. The data for each specimen included a combination of the fungal species’ identity, the collection site location and date of collection. For example, two fungi of the same species collected at the same location on different dates are treated here as two separate specimens. Several attributes for each specimen were recorded when available. These attributes include the country, region and coordinates of the collection site, date of collection, insect host, publication year, authors and Digital Online Identifier. Collection site coordinates were manually validated by comparing the provided collection site coordinates to the provided collection site description. Additionally, up-to-date fungal taxonomy information including the phylum, class, order, family and genus of the fungi were obtained from the GBIF database (15). For specimens with provided coordinates, collection year and collection month, the monthly average precipitation and the minimum and maximum monthly temperatures were extracted from WorldClim.org using the specific date and location of each collection record when data were available (17). The average monthly temperature was calculated as the mean of the minimum and maximum monthly temperatures. For specimens with recorded host associations, the insect host family and common name were obtained from the NCBI taxonomy database (18).

### User interface creation

The CIGAF website was created using R Shiny. Interactive visualizations within the site were generated in R using the *ggplot, ggbeeswarm, plotly, leaflet, heatmaply* and *collapsibletree* packages (19–21). To facilitate the dissemination of information, there is a link-out feature on the taxonomy tab, which directs the user to information web pages of each species hosted at https://keys.lucidcentral.org/keys/v4/trichomycetes/keys/index.html (10). User input panels were constructed and embedded into the site using Google Survey tools. The CIGAF web application underwent several iterative rounds of user experience testing with experts and nonexpert testers to refine and improve the user interface.

## Results and discussion

### Data types and database overview

**Figure 1.**
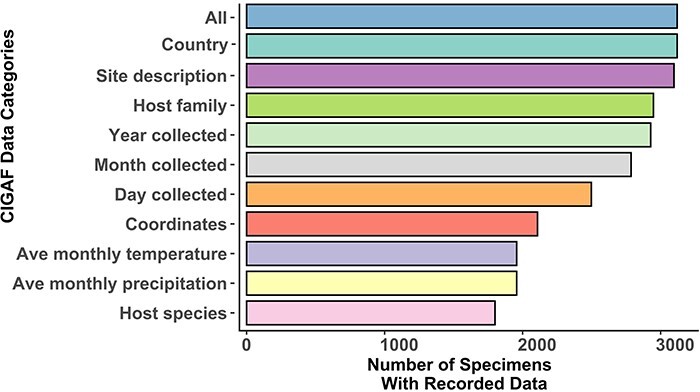
A bar chart of data associated with collection records within the CIGAF database. ‘All’ is the total number of records within the database, ‘Country’ includes records with a recorded country of collection and ‘Site description’ includes entries with a detailed collection site description. ‘Year collected’, ‘Month collected’ and ‘Day collected’ are entries with time of collection data recorded at each level of specificity. ‘Host family’ is the number of specimens with their host identified at the insect family level, while ‘Host species’ records have their host identification at the species level. ‘Coordinates’ is the number of entries with associated latitude and longitude (from the original publication, not extrapolated from the site description). ‘Ave monthly precipitation’ and ‘Ave monthly temperature’ are averages extrapolated from the WorldClim database (17) for records that have associated coordinates, year of collection and month of collection.

**Figure 2.**
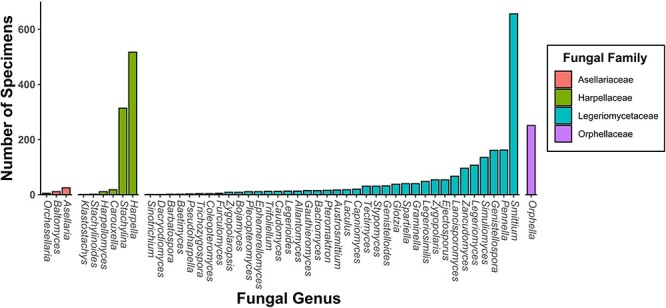
A bar chart of specimen records within the CIGAF database by genus. The bars are colored by fungal family and are ordered from lowest to highest prevalence within each category.

**Figure 3.**
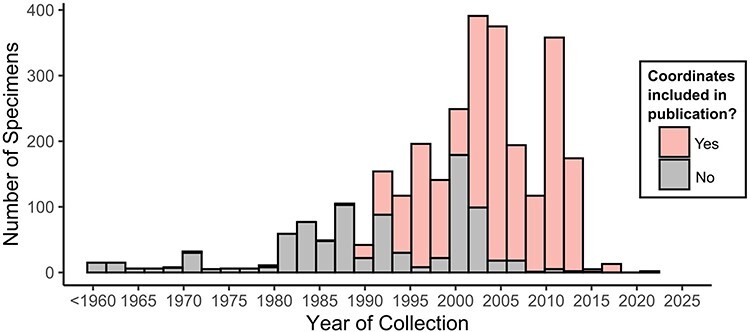
Histogram of specimens within the CIGAF database by year. Histogram bars are stacked and colored pink if coordinates were recorded in the original publication for the associated specimens.

**Figure 4.**
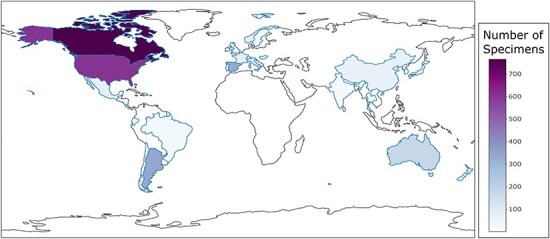
Country-level choropleth map of trichomycetes generated using the CIGAF user interface. The legend shows the trichomycetes specimen intensity, with the darker purple color indicating the larger number of associated collection records. Countries that show a white color without a defined blue outline have zero collection records within the database.

**Figure 5.**
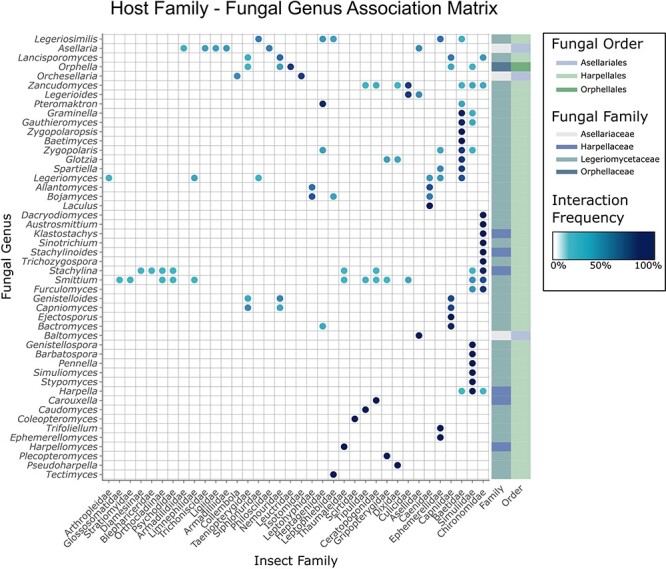
Matrix of trichomycetes–insect interactions generated using the CIGAF web application. Insect hosts at the family level are shown along the *x*-axis, and fungal genera are shown along the *y*-axis. The darker the dot at any row–column intersection, the higher the prevalence (number of records containing the indicated trichomycetes–insect interaction). Fungal genera (rows) are clustered by interaction pattern similarity such that genera with similar interaction patterns are next to each other. Insect hosts are ordered alphabetically. The rectangle array at the end of each row indicates the fungal family and fungal order to which the corresponding fungal genus belongs.

The following figures summarize the data records within the CIGAF database. Figure 1 summarizes the data types available within the CIGAF database and the number of specimens for which the associated data are available. Figure 2 shows the taxonomic diversity of the database, which includes 3120 specimens, 289 species, 48 genera, 4 families and 3 orders of insect gut–associated fungi. Figure 3 shows a temporal distribution of collection records, which spans from 1929 to 2022. Figure 4 shows the geographic range of collections, indicating that although collections are highest in North America, trichomycetes have a cosmopolitan distribution with a wide range of latitudes from Norway to the French Antarctic Islands. Figure 5 presents the trichomycetes–insect associations in an interaction matrix with trichomycete fungi at the genus level and insect hosts at the family level, demonstrating the broad diversity of trichomycetes, and their host specificity vary among taxa.

### CIGAF web application

**Figure 6.**
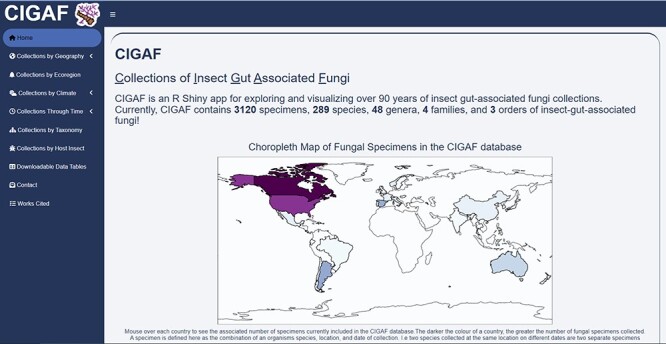
The CIGAF website homepage showing a descriptive overview of the site contents, with the expanding tab list on the left.

The CIGAF interface, as shown in Figure 6, was created to enable user-friendly access and exploration of the CIGAF database. This application contains tools that can facilitate the investigation of several ecological perspectives regarding trichomycetes prevalence and insect associations. When the user selects the ‘Collections by Geography’ tab, they can choose to focus on specific collection sites, country-level diversity or country-level range of a taxon of interest. The ‘Collections by Ecoregion’ tab provides a bar chart breakdown of specimen count by realm, biome or ecoregion based on the Resolve Ecoregions and Biomes Map for all taxa or a taxon of interest. The ‘Collections by Climate’ tab creates a point or box plot of average monthly temperature or precipitation, grouping specimens along the *x*-axis at a user-selected taxonomic level. The ‘Collections by Time’ tab includes histogram plots of specimens by collection or publication date, where the date range and color breakdown of the histogram bars are selected by the user. The ‘Collections by Taxonomy’ tab has an interactive, expanding hierarchical tree, breaking down collections taxonomically from their order to species level. When a species level node is selected by the user, a link-out button appears containing an image and description of the species when available. The ‘Collections by Host Insect’ tab includes a large association matrix showing recorded associations as a percentage of all associations of a given fungal genus. There are two stacked bar plot visualizations that show a user-selected fungal taxonomy level’s insect associations and the specimen count or percentage, respectively. The ‘Downloadable Data Tables’ tab includes a searchable, subsettable and downloadable data table of the CIGAF database. The ‘Contact’ tab includes Google Forms for submission of new specimens or errors to the CIGAF database curators, enabling future expansion of the database.

## Technical validation

Records with associated collection site information were individually validated to ensure that the provided coordinate location plausibly matched the associated collection site descriptions. The CIGAF application was tested for ease of use with several experts and nonexpert volunteers who completed a set of sample tasks. These tasks included identifying the range and number of specimens of a given fungal species, genus or family, providing an overview of the host associations of a given fungal species, and timeline of collections within a specific country. The design, in-application texts and plots produced within the application were improved based on their input to create CIGAF version 1.

## Usage notes

As depicted in the choropleth map of collected trichomycetes by region, collection levels are highest within North America (Figure 4). This introduces some potential biases into the data. Therefore, it is recommended that the target question is considered carefully such that an appropriate subset of the data included in CIGAF can be used for the study. Limiting the specimens in an exploratory ecological study to those that have coordinates within a specific ecoregion will help to minimize these biases. The inconsistent collection efforts across the globe can also be informative for directing future collections, such that regions with low specimen counts could be prioritized over regions that are already well sampled.

The CIGAF database will expand continuously as new collection records are published or added via the data submission tab available on the CIGAF webpage (via Contact tab). To facilitate the best integration of novel records into the database, four recommendations are provided for the documentation of future collection efforts:

- Provide coordinates in decimal format for each collection site [e.g. (43.7831, −79.1875) instead of (43°46′59″N, 79°11′15″W)]. This provides an accurate account of the collection site while avoiding issues with special characters when automatic text processing software is used.
- Record the specific date of collection. This will facilitate comparative studies focusing on the long-term effects of temporal climatic, environmental or biological phenomena on trichomycetes and insect associations.
- Record the insect hosts to the finest taxonomic level, minimally to the family level. This will enable broader scale investigations such as host specificity and potential host-jumping events of trichomycetes.
- Include precise site descriptions with comments on the estimated water flow rate, presence of vegetation, pollution and other ecological factors where possible. This will enable ecological research of trichomycete fungi and associated insects including comparative studies using datasets collected at distant sites with similar ecological features.

Overall, we are excited to announce the launch of CIGAF, a cutting-edge interactive research platform for the globally distributed trichomycete fungi and their associated insect hosts. This new platform provides a wide array of informative datasets and features that will undoubtedly elevate research and education of trichomycetes as well as fungus–insect symbiosis. CIGAF is designed to make the study of trichomycete fungi and their associated insect hosts easier and more efficient. This platform offers a range of benefits for researchers and educators. First, it allows them to analyze both geographic distributions and insect associations in one place. Additionally, the platform provides new tools that can help researchers identify gaps in their knowledge and generate informed hypotheses about trichomycete fungi and symbiosis more broadly. Meanwhile, educators can use the platform for teaching purposes and to help students understand this complex area of research. We believe that CIGAF has the potential to revolutionize the study of trichomycete fungi and expect more in-depth studies will follow through the use of this platform.

## Code availability

The code for the CIGAF Shiny Application has been deposited at https://github.com/WangLab-UToronto/CIGAF.git. The website and CIGAF database are hosted by the University of Toronto and are accessible at http://cigaf.eeb.utoronto.ca.
